# Impact of a phased transition model on Advanced HIV Disease outcomes: A pre- and post-implementation evaluation study in Malawi

**DOI:** 10.1371/journal.pgph.0006348

**Published:** 2026-10-23

**Authors:** Thulani Maphosa, Rhoderick Machekano, Lise Denoeud Ndam, Lucky Makonokaya, Lloyd Chilikutali, Louiser Upile Kalitera, Eddington Matiya, Allan Mayi, Reuben Musarandega, Bilal Wilson Matola, Aida Yemane Berhan, Allan Ahimbisibwe, Appolinaire Tiam

**Affiliations:** 1 Elizabeth Glaser Pediatric AIDS Foundation, Lilongwe, Malawi; 2 Elizabeth Glaser Pediatric AIDS Foundation, Washington, District of Columbia, United States of America; 3 Elizabeth Glaser Pediatric A.I.D.S. Foundation, Geneva, Switzerland; 4 Department of HIV and AIDS, Ministry of Health, Lilongwe, Malawi; 5 George Washington University, Washington, District of Columbia, United States of America; Federal University of Mato Grosso do Sul, BRAZIL

## Abstract

To promote sustainability and strengthen national ownership of Advanced HIV Disease (AHD) services, a transition was implemented across 22 health facilities in Central Malawi. This transition involved shifting responsibility for key AHD program elements from partner-led implementation to full Ministry of Health (MoH) leadership. This evaluation assessed the impact of this transition on diagnostic coverage, TB preventive therapy (TPT) uptake, and 12-month survival outcomes. A retrospective cohort study included all children and adults enrolled in AHD care during the pre-MoH transition (January 2020–December 2021) and post-MoH transition (January 2023–December 2024) periods. Eligibility was defined by national AHD criteria: CD4 count <200 cells/mm³, WHO stage 3 or 4 disease, or age < 5 years. Data were abstracted from clinical records and routine facility registers. Kaplan–Meier survival curves, Cox proportional hazards, and Fine and Gray competing risk models were used to evaluate 6 and 12-month mortality and retention as primary outcomes. A total of 1,044 AHD clients were included (553 pre-transition; 491 post-transition). Median age increased post-transition (35.9 to 38.5 years, p < 0.001). CD4 testing uptake declined (80.7% to 46.0%, p < 0.001), while WHO staging and TB diagnostic coverage improved. TB diagnoses decreased (44.5% to 31.2%, p = 0.002). TPT uptake dropped from 46.4% to 31.6% (p < 0.001). Twelve-month mortality declined significantly from 9.4% to 5.5% (adjusted hazard ratio [aHR] = 0.59, 95% CI: 0.37–0.94, p = 0.026). Retention in care remained stable (HR = 0.86, 95% CI: 0.62–1.20, p = 0.384). Transitioning AHD services to MoH leadership was associated with sustained key program outcomes and a significant reduction in mortality. Continued mentorship and government ownership appeared to support these outcomes, although the pre-post design precludes causal inference. Declines in CD4 testing and TPT coverage highlight the need to strengthen diagnostics and integrate preventive care. These findings support scaling nationally led AHD models in high-burden HIV settings.

## Background

Despite substantial progress in global HIV care and treatment, Advanced HIV Disease (AHD) remains a persistent contributor to AIDS-related morbidity and mortality, particularly in sub-Saharan Africa [1–3]. Nearly one in three people starting antiretroviral therapy (ART) in the region presents with advanced disease, defined by the WHO as a CD4 count <200 cells/mm³, WHO stage 3 or 4 disease, or age < 5 years. In Malawi, a country with a high HIV burden and longstanding ART programs, approximately 50% of adult ART clients with AHD are not diagnosed or managed appropriately, especially in decentralized settings [3–5]. This gap results in late detection of opportunistic infections such as tuberculosis (TB), cryptococcal meningitis, and severe bacterial infections, which account for the majority of preventable HIV-related deaths [1,3,6–8].

In response, the World Health Organization developed the AHD care package, recommending a standardized approach that includes rapid CD4 testing, TB-lipolipoarabinomannan (LAM) and cryptococcal antigen (CrAg) screening, and expedited ART initiation [2]. However, most AHD program implementations have been partner-led and concentrated at central hospitals, creating a sustainability gap in health system ownership, capacity, and integration. In Malawi, between 2020 and 2021, Elizabeth Glaser Pediatric AIDS Foundation (EGPAF), a non-governmental organization (NGO), introduced a hub-and-spoke model at 22 health facilities, providing diagnostics, training, mentorship, and treatment support for AHD. While this initiative yielded improvements in detection and outcomes, questions remained about how to sustain these interventions as donor support wanes and national health systems are expected to assume full ownership [9–11].

To address this, a structured transition model was developed and implemented beginning in 2022 to transfer full responsibility for AHD service delivery to the Malawi Ministry of Health (MoH). The transition encompassed key domains of service delivery, including commodity management, health worker training, mentorship, data integration, and supervision, shifting leadership from EGPAF to district health offices and national HIV program units. This transition was critical to institutionalizing AHD care within existing MoH systems, ensuring the continuity and sustainability of lifesaving interventions [11,12].

We conducted an evaluation to document the feasibility, effectiveness, and outcomes of transitioning a complex HIV service delivery model from partner-led to nationally owned. Most evaluations focus on clinical outcomes or short-term donor performance metrics. Few have rigorously examined what happens to program coverage, quality, and patient outcomes after such a transition, particularly for high-risk populations like those with AHD. By understanding these dynamics, national HIV programs and development partners can design more resilient systems and optimize resource allocation to sustain progress toward the UNAIDS 95-95-95 goals and reduce AIDS-related mortality [2].

To assess the implementation and outcomes of the AHD program transition in Malawi, we evaluated [1] whether health service coverage, clinical outcomes, and patient retention among clients with AHD changed significantly between the pre-transition (2020–2021) and post-transition (2022–2023) periods, and [2] the extent to which the MoH successfully assumed responsibility for program delivery, supervision, and data integration across transition domains.

## Methods

### Ethics statement

Data collection was limited to secondary data abstraction from routinely collected and documented data as part of the AHD standard medical care of the project, with no direct interaction between the program evaluation staff and study participants. This study and all the procedures involved in collecting and analyzing routine program data imposed minimal risk to participants; therefore, the Institutional Review Boards granted a waiver of consent.

Ethical clearance was obtained from the National Health Science Research Committee on 21/06/2021. The Advarra Institutional Review Board reviewed and approved the study on 6 October 2021 (protocol number Pro00057717). All study methods were performed in accordance with relevant guidelines and regulations.

### Study design and setting

We conducted a non-randomised, cluster-based pre-post implementation study to evaluate the outcomes of transitioning an optimized AHD care model from a partner-led approach to full MoH ownership in Malawi. The intervention was implemented in three districts, Ntcheu, Dedza, and Mchinji, in the Central Region of Malawi, selected due to their high HIV burden and early adoption of the AHD care package. The evaluation study was implemented across 22 purposively selected health facilities, comprising 10 hub hospitals and 12 satellite spoke sites. The intervention was implemented in three temporal phases aligned with the progressive transition of responsibilities from partner-led to government-led management. Table 1 shows that during the pre-transition phase (January 2020 to December 2021), the program was led by the EGPAF, which provided full implementation support. This was followed by the transition phase (January to December 2022), during which EGPAF and the MoH jointly coordinated activities to facilitate capacity-building and systems integration. Finally, in the post-transition phase (January 2023 to December 2024), full program leadership and operations were assumed by the MoH. Client outcomes across all phases were assessed using a retrospective cohort design with follow-up extending up to 12 months after enrolment into AHD care.

**Table 1:** Intervention description for AHD program transition matrix.

Domain	Pre-Transition (2020–2021)	Transition Phase (2022)	Post-Transition (2023–2024)
**Program Leadership**	Externally driven implementation led by EGPAF	Co-governance established between EGPAF and MoH	Full institutional ownership and leadership by MoH
**AHD Diagnostic Commodities**	Parallel procurement and distribution managed by EGPAF	Strengthened stock visibility through integration with MoH LMIS	Fully integrated procurement within national supply chain systems
**Health Workforce Capacity**	Facility-based training led by EGPAF	Embedded mentorship co-delivered with MoH teams	Institutionalized MoH-led CME and supportive supervision systems
**Service Delivery Model**	Centralized delivery at 10 hub facilities	Decentralization to spoke facilities with partner support	District-wide scale-up across 22 facilities under MoH
**Monitoring & Evaluation Systems**	Parallel AHD registers and reporting systems	Progressive alignment with national reporting frameworks	Full integration into the national DHIS2 architecture
**Guidelines & SOPs**	Partner-developed job aids and protocols	Joint revision and contextualization with MoH input	Nationally endorsed and standardized AHD SOPs
**Mentorship & Technical Assistance**	Intensive partner-led technical assistance	Transition to joint mentorship through MoH TWGs	MoH-led mentorship systems with minimal external support
**Community Linkages**	Limited, facility-based patient identification	Pilot community referral pathways initiated	Integration with CHWs and Expert Clients within national programs
**Evidence Generation & Use**	Partner-driven operational research	Structured dissemination to MoH TWGs and stakeholders	Routine MoH-led data review and evidence-informed decision-making
**Quality Improvement Systems**	Partner-led QI data collection and review	Introduction of joint QI coaching and learning platforms	Institutionalized MoH-led DQA and continuous QI systems

EGPAF, Elizabeth Glaser Pediatric AIDS Foundation; MoH, Ministry of Health; LMIS, Laboratory Management Information System; CME, Continuing Medical Education; SOP, Standard Operating Procedure; TWG, Technical Working Group; CHW, Community Health Worker; DQA, Data Quality Assessment; QI, Quality Improvement.

### Study population and eligibility criteria

The study population comprised all children and adults enrolled in AHD care at the 22 study sites during the pre- and post-transition phases (excluding 2022, when transition was ongoing). The decision to exclude the 2022 transition year was made a priori to avoid mixing exposure states within a single analytical cohort. During 2022, program leadership was jointly shared between EGPAF and the MoH, and the pace and completeness of responsibility transfer varied substantially across the 22 facilities and nine transition domains (Table 1). Including clients enrolled during this period would introduce heterogeneous and unmeasured exposure, complicating the interpretation of phase-specific outcomes. This approach is consistent with pre-post evaluation designs that use clean comparison periods to reduce contamination bias. During 2022, the transition activities included structured co-mentorship between EGPAF and district health teams, joint revision of national AHD SOPs, and progressive handover of commodity procurement – activities that reflected the dynamic nature of the transition and the rationale for its exclusion from the outcome analysis. Eligibility followed national AHD criteria, which included people living with HIV with a CD4 count of less than 200 cells/mm³, a WHO clinical stage 3 or 4 illness, or being under 5 years of age, regardless of clinical or immunologic status. Clients were excluded if they lacked documented AHD-defining criteria or had incomplete clinical records that could not be verified. In total, 1,044 clients were included in the final analysis, 553 during the pre-transition phase and 491 during the post-transition phase.

### Intervention description

The intervention involved the structured transition of responsibilities across nine key domains: program leadership, commodity procurement, human resource capacity, service site coverage, monitoring and evaluation, guidelines and SOPs, mentorship and technical assistance, community linkages, and quality improvement.

The pre-transition phase was characterized by intensive EGPAF support, including partner-led procurement, training, and parallel monitoring systems. During the transition phase, joint planning and undertaken. In the post-transition phase, all program components were fully managed by the MoH, with integration into national systems such as DHIS2 and health facility-led mentorship. Table 1.

### Sample size and power

Assuming a baseline 6-month mortality of 12%, we calculated that a minimum of 440 clients per study phase would be required to detect a 30% relative reduction in mortality (α = 0.05, power = 80%). The achieved sample (n = 1,044) provided adequate statistical power for subgroup and survival analyses.

### Outcomes and follow-up

The primary outcomes for this study were all-cause mortality and retention through 12 months following enrolment in AHD care. Secondary outcomes included retention in care at 3, 6, and 12 months; uptake of TB preventive therapy (TPT) among eligible clients; diagnostic coverage, including CD4 testing, CrAg screening, and GeneXpert testing for TB; and the frequency of opportunistic infections at the time of presentation. All clients enrolled in the cohort were followed for up to 12 months from the date of AHD enrolment to assess these outcomes.

### Data collection and management

Data were retrospectively extracted from multiple sources, including the AHD register, ART patient files, laboratory logs, and the national EMR system. ART numbers served as unique identifiers to triangulate and de-duplicate records. Data abstraction was conducted using Open Data Kit (ODK) tablets, and datasets were exported into Stata 17.0 (StataCorp, College Station, TX) for analysis. Data abstraction during both pre- and post-transition phases was performed by trained research assistants who received standardized training on AHD register completion and data abstraction protocols prior to data collection. The same cadres of data collectors were involved across both phases to ensure consistency. Pre-transition data collection commenced one month before the close of the pre-transition period to enable timely data lock, whereas post-transition data collection began ten months before the end of the post-transition period to allow sufficient follow-up time for 12-month outcome ascertainment. These differences in abstraction timing reflect the study’s longitudinal design requirements rather than methodological inconsistency.

### Statistical analysis

Descriptive statistics were used to summarize demographic and clinical characteristics. Pre-post differences were assessed using Chi-square or Fisher’s exact tests for categorical variables and Wilcoxon rank-sum tests for non-parametric continuous variables.

Kaplan–Meier survival curves and the log-rank test were used to compare time to death and retention outcomes. Cox proportional hazards models and Fine-Gray subdistribution hazard models were applied to assess the effect of the intervention while adjusting for potential confounders and accounting for competing risks, such as treatment discontinuation and transfer out. Results are presented as hazard ratios (HRs) and sub-distribution hazard ratios (sHRs) with 95% confidence intervals. A p-value <0.05 was considered statistically significant. Multivariable models were built using a purposive selection approach, including variables identified a priori as clinically or programmatically relevant: transition phase, age group, sex, ART treatment status, inpatient enrolment status, AHD eligibility criterion (CD4 vs. WHO staging), and WHO stage. The proportional hazards assumption was evaluated graphically using Schoenfeld residuals and complementary log-log plots; no significant violations were identified. Missing data were handled using complete-case analysis, given that missingness was predominantly in laboratory variables (notably CD4 count), which were themselves outcome indicators. The extent and patterns of missingness are reported in the results. Facility-level clustering was accounted for by including facility as a stratification variable in the Cox models, given the study’s cluster-based design.

## Results

Among the 1,044 clients enrolled in AHD care, baseline characteristics varied significantly between the pre- and post-transition periods (Table 2). There was a comparable sex distribution across phases (p = 0.40), with females constituting slightly more than half of the population. However, age distributions differed significantly (p = 0.007), with a greater proportion of older clients (≥50 years) enrolled post-transition. The mean age was also significantly higher post-transition (35.9 vs. 38.5 years, p < 0.001).

**Table 2:** Baseline characteristics of clients by phase of AHD program implementation.

Characteristic	Pre-Transition (n=553)	Post-Transition (n=491)	Total (N=1,044)	p-value
**Sex**	**0.397**			
Male	278 (50.3%)	233 (47.5%)	511 (48.9%)	
Female	275 (49.7%)	258 (52.5%)	533 (51.1%)	
**Age group**				0.007**
<5 years	39 (7.1%)	16 (3.3%)	55 (5.3%)	
5–14 years	15 (2.7%)	16 (3.3%)	31 (3.0%)	
15–24 years	55 (9.9%)	48 (9.8%)	103 (9.9%)	
25–49 years	351 (63.5%)	294 (59.9%)	645 (61.8%)	
≥50 years	93 (16.8%)	117 (23.8%)	210 (20.1%)	
**Current age, mean (SD)**	35.9 (15.4)	38.5 (15.7)	37.1 (15.6)	<0.001***
**CD4 Count Done**				<0.001***
No	107 (19.3%)	265 (54.0%)	372 (35.6%)	
Yes	446 (80.7%)	226 (46.0%)	672 (64.4%)	
**CD4 count result, median (IQR)**	133.5 (77.0)	182.0 (199.0)	171.0 (103.0)	<0.001***
**AHD eligibility: CD4 <200 cells/mm³**				<0.001***
No	187 (33.8%)	271 (55.2%)	458 (43.9%)	
Yes	366 (66.2%)	220 (44.8%)	586 (56.1%)	
**AHD eligibility: WHO stage 3/4**				<0.001***
No	306 (55.3%)	181 (36.9%)	487 (46.6%)	
Yes	247 (44.7%)	310 (63.1%)	557 (53.4%)	
**AHD eligibility: Children <5 years**				**0.128**
No	515 (93.1%)	469 (95.5%)	984 (94.3%)	
Yes	38 (6.9%)	22 (4.5%)	60 (5.7%)	
**New on ART**				0.042*
No	169 (37.9%)	67 (29.6%)	236 (35.1%)	
Yes	277 (62.1%)	159 (70.4%)	436 (64.9%)	
**High Viral Load (>1,000 copies/mL) before AHD enrolment**				**0.337**
No	412 (92.4%)	214 (94.7%)	626 (93.2%)	
Yes	34 (7.6%)	12 (5.3%)	46 (6.8%)	
**Defaulted in care before AHD enrolment**				**1.000**
No	392 (87.9%)	199 (88.1%)	591 (87.9%)	
Yes	54 (12.1%)	27 (11.9%)	81 (12.1%)	
**Inpatient care at AHD enrolment**				0.035*
No	421 (94.4%)	222 (98.2%)	643 (95.7%)	
Yes	25 (5.6%)	4 (1.8%)	29 (4.3%)	
**Treatment Status at AHD enrolment**				0.008**
Treatment naïve	419 (75.8%)	405 (82.7%)	824 (79.0%)	
Treatment experienced	134 (24.2%)	85 (17.3%)	219 (21.0%)	
**Last ARV regimen taken before AHD enrolment**				**0.489**
NNRTI	106 (79.1%)	74 (87.1%)	180 (82.2%)	
PI	13 (9.7%)	6 (7.1%)	19 (8.7%)	
INSTI	12 (9.0%)	4 (4.7%)	16 (7.3%)	
Other	3 (2.2%)	1 (1.2%)	4 (1.8%)	
**Time on ART, mean months (SD)**	50.8 (31.9)	59.0 (38.5)	53.9 (34.7)	<0.001***

Values are presented as n (%), unless otherwise specified. p-values derived from Chi-square test, Fisher’s exact test, or t-test as appropriate. *p<0.05, **p<0.01, ***p<0.001. ART, antiretroviral therapy; AHD, advanced HIV disease; IQR, interquartile range; SD, standard deviation; NNRTI, non-nucleoside reverse-transcriptase inhibitor; PI, protease inhibitor; INSTI, integrase strand-transfer inhibitor.

CD4 testing coverage declined pre- to post-transition (80.7% vs. 46.6%, respectively, p < 0.001), although the median CD4 count was higher in the post- than pre-transition group (134 vs. 182, respectively, p < 0.001) (Table 2). A higher proportion of clients met AHD eligibility based on CD4 < 200 during the pre- than post-transition period (66.2% vs. 44.8%, respectively, p < 0.001), while more were classified as AHD based on WHO stage 3/4 in the post- than pre-transition phase (44.7% vs. 63.1%, respectively, p < 0.001). The proportion of new ART initiates was higher post-transition than pre-transition phase (62.1% vs 70.4%, p = 0.04), and fewer clients at post-transition were enrolled as inpatients (5.6% vs. 1.8%, p = 0.0348). Treatment-naïve clients were more in the post- than pre-transition phase (75.8% vs. 82.7%, respectively, p = 0.008), and the mean time on ART was significantly longer in the post-transition (50.8 vs. 59.0 months, p < 0.001). No significant differences were observed in viral load suppression, ART defaulting before AHD enrolment, or prior ART regimens.

Table 3 highlights key differences in the clinical presentation of clients across the phases of the AHD program. TB remained the most frequently diagnosed opportunistic infection but declined significantly from 44.5% in the pre-transition period to 31.2% post-transition (p  =  0.002). Similarly, significant reductions were observed in cases of candidiasis (3.4% vs. 0.0%, p  =  0.005) and severe weight loss (12.5% vs. 5.0%, p  =  0.003). In contrast, bacterial infections increased significantly in the post- compared with the pre-transition phase (6.8% vs 12.5%, p = 0.04). Other conditions, such as cryptococcal meningitis, Kaposi sarcoma, and severe pneumonia, were less common and did not differ significantly between phases. Among WHO stage 3 and 4 defining conditions documented post-transition, pulmonary TB remained the most common, followed by severe bacterial infections and severe pneumonia; oral candidiasis, among the most frequently recorded stage 3 conditions pre-transition, was absent post-transition. The overall decline in diagnosed OIs post-transition should be interpreted with caution: it may reflect genuine improvement in clinical status at presentation (consistent with earlier enrolment and less advanced disease), but may also partly reflect reduced screening sensitivity where OI-specific protocols were less consistently applied without intensive partner mentorship.

**Table 3:** Opportunistic Infections and Related Signs and Symptoms by Phase of AHD Program Implementation.

Opportunistic Infection / Clinical Condition	Pre-Transition	Post-Transition	Total	p-value
**Tuberculosis**				0.002**
Yes	117 (44.5%)	87 (31.2%)	204 (37.6%)	
No	146 (55.5%)	192 (68.8%)	338 (62.4%)	
**Cryptococcal Meningitis**				**0.118**
Yes	17 (6.5%)	9 (3.2%)	26 (4.8%)	
No	246 (93.5%)	270 (96.8%)	516 (95.2%)	
**Kaposi Sarcoma**				**0.143**
Yes	10 (3.8%)	4 (1.4%)	14 (2.6%)	
No	253 (96.2%)	275 (98.6%)	528 (97.4%)	
**Bacterial Infection**				0.037*
Yes	18 (6.8%)	35 (12.5%)	53 (9.8%)	
No	245 (93.2%)	244 (87.5%)	489 (90.2%)	
**Candidiasis**				0.005**
Yes	9 (3.4%)	0 (0.0%)	9 (1.7%)	
No	254 (96.6%)	279 (100.0%)	533 (98.3%)	
**Chronic/Severe Cough**				**0.505**
Yes	0 (0.0%)	2 (0.7%)	2 (0.4%)	
No	263 (100.0%)	277 (99.3%)	540 (99.6%)	
**Herpes Zoster**				**0.226**
Yes	3 (1.1%)	0 (0.0%)	3 (0.6%)	
No	260 (98.9%)	279 (100.0%)	539 (99.4%)	
**Severe Diarrhoea**				**0.143**
Yes	10 (3.8%)	4 (1.4%)	14 (2.6%)	
No	253 (96.2%)	275 (98.6%)	528 (97.4%)	
**Fever**				**0.218**
Yes	9 (3.4%)	4 (1.4%)	13 (2.4%)	
No	254 (96.6%)	275 (98.6%)	529 (97.6%)	
**Severe Weight Loss or Wasting**				0.003**
Yes	33 (12.5%)	14 (5.0%)	47 (8.7%)	
No	230 (87.5%)	265 (95.0%)	495 (91.3%)	
**Severe Malnutrition**				**0.325**
Yes	2 (0.8%)	6 (2.2%)	8 (1.5%)	
No	261 (99.2%)	273 (97.8%)	534 (98.5%)	
**Severe Pneumonia**				**0.074**
Yes	3 (1.1%)	11 (3.9%)	14 (2.6%)	
No	260 (98.9%)	268 (96.1%)	528 (97.4%)	
**Other Opportunistic Conditions**				—
Yes	87 (33.1%)	55 (19.7%)	142 (26.2%)	

Values are presented as n (%). Denominators vary by condition as data were available only for clients screened for each opportunistic infection. p-values derived from the Chi-square test or Fisher’s exact test, as appropriate. *p<0.05, **p<0.01, ***p<0.001. AHD, advanced HIV disease.

Of the 1,044 clients enrolled, 80.7% were eligible for TPT. Uptake was significantly higher in the pre-transition phase (46.4%) compared to the post-transition phase (31.6%) (p  <  0.001), indicating reduced coverage following the transition (Table 4).

**Table 4:** TB preventive therapy among eligible patients by phase of AHD program implementation.

Indicator	Pre-Transition (n=553)	Post-Transition (n=491)	Total (N=1,044)	p-value
**Total clients enrolled (N)**	**553**	**491**	**1,044**	
**TPT Eligibility**				<0.001***
Eligible (Yes)	476 (86.1%)	367 (74.7%)	843 (80.7%)	
Not eligible (No)	77 (13.9%)	124 (25.3%)	201 (19.3%)	
**Received TB Preventive Therapy (TPT) (among eligible clients)**				<0.001***
Yes	221 (46.4%)	116 (31.6%)	337 (40.0%)	
No	255 (53.6%)	251 (68.4%)	506 (60.0%)	

Values are presented as n (%), unless otherwise specified. p-values derived from the Chi-square test. TPT denominators are based on eligible clients only (n=476 pre-transition; n=367 post-transition; N=843 total). ***p<0.001. AHD, advanced HIV disease; TPT, tuberculosis preventive therapy.

Treatment outcomes were comparable across phases, with 70.2% of clients alive on treatment in the pre-transition phase and 71.0% in the post-transition phase (Table 5A). However, mortality was lower post-transition (9.4% vs. 5.5%), and survival analysis showed significantly reduced mortality risk in the post-transition phase at 3, 6, and 12 months (Table 5B) and (Fig 1), supported by Cox regression (HR = 0.59, 95% CI: 0.37–0.94, *p* = 0.026) and Fine and Gray models (HR = 0.58, 95% CI: 0.36–0.93, *p* = 0.024) (Table 5C). While out-of-care risk estimates slightly favored the post-transition phase at each time point (Table 5D), the difference was not statistically significant (HR = 0.86, 95% CI: 0.62–1.20, *p* = 0.384) (Table 5E) and (Fig 2).

**Table 5:** Treatment outcomes and survival by pre- and post-transition period.

A. Treatment Outcomes by Phase of AHD Program Implementation					
Outcome	Pre-Transition (n=553)	Post-Transition (n=491)	p-value		
**Alive on treatment**	388 (70.2%)	348 (71.0%)	0.063		
Died	52 (9.4%)	27 (5.5%)			
Transfer Out	83 (15.0%)	81 (16.5%)			
Stopped Treatment	2 (0.4%)	0 (0.0%)			
Defaulted	28 (5.1%)	34 (6.9%)			
B. Estimated Mortality Risk at 3, 6, and 12 Months (Kaplan–Meier)					
**Phase**	**Time (months)**	**At Risk**	**Deaths / Events**	**Failure (%)**	**95% CI**
**Pre-transition**	3	442	9	6.3%	4.5–8.9%
	6	411	12	8.3%	6.1–11.1%
	12	94	—	12.1%	9.2–15.8%
**Post-transition**	3	397	3	4.1%	2.6–6.4%
	6	373	4	4.8%	3.2–7.3%
	12	67	2	6.0%	4.1–8.8%
C. Comparison of Mortality Risk: Pre- vs. Post-Transition					
**Model**	**Hazard Ratio (aHR)**	**95% Confidence Interval**	**p-value**		
Cox proportional hazards regression	0.59	0.37–0.94	0.026*		
Fine & Gray competing-risk regression	0.58	0.36–0.93	0.024*		
D. Estimated Out-of-Care Risk at 3, 6, and 12 Months (Kaplan–Meier)					
Phase	Time (months)	At Risk	Deaths / Events	Failure (%)	95% CI
**Pre-transition**	3	442	16	9.0%	6.8–11.9%
	6	411	18	12.4%	9.8–15.6%
	12	94	—	17.6%	14.2–21.6%
**Post-transition**	3	397	10	8.6%	6.3–11.5%
	6	373	9	10.9%	8.3–14.2%
	12	67	3	13.3%	10.4–16.9%
**E. Comparison of Retention (Out-of-Care) Risk: Pre- vs. Post-Transition**					
**Model**	**Hazard Ratio (aHR)**	**95% Confidence Interval**	**p-value**		
Cox proportional hazards regression	0.86	0.62–1.20	0.384		

Values are presented as n (%), unless otherwise indicated. CI, confidence interval; aHR, adjusted hazard ratio. Panel A p-value is for the overall distribution of outcomes derived from the Chi-square test. Panels B and D present Kaplan–Meier cumulative failure estimates; a dash (—) in the Failures column indicates 0 events at 12 months (Panel B, pre-transition) and 0 events at 12 months (Panel D, pre-transition). Panels C and E present adjusted hazard ratios from Cox proportional hazards and Fine & Gray competing-risk regression, adjusted for age group, sex, ART treatment status, inpatient enrolment, and AHD eligibility criterion. *p < 0.05.

**Fig 1:**
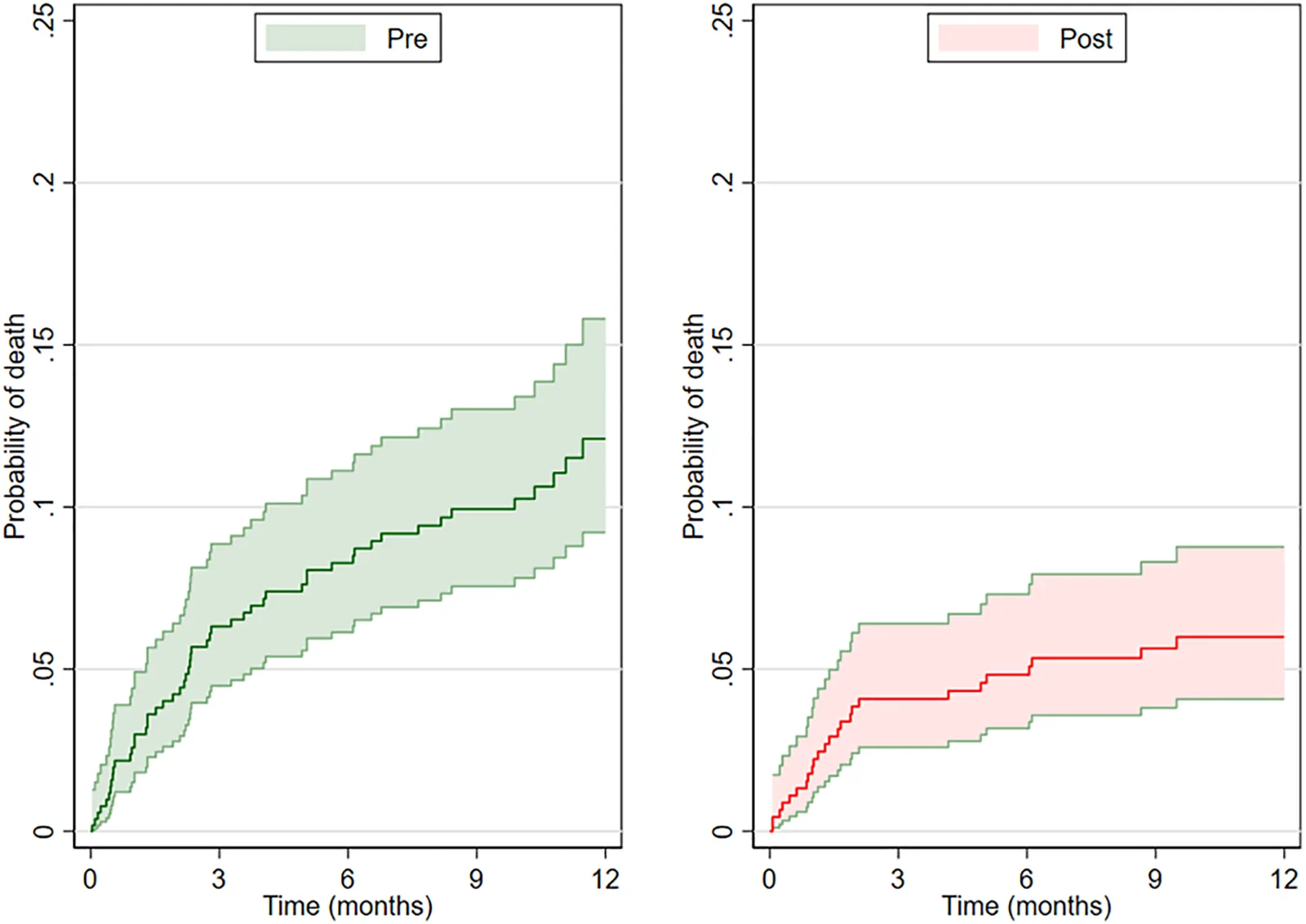
Twelve-month cumulative probability of death among clients with advanced HIV disease before and after transition to Ministry of Health-led service delivery.

**Fig 2:**
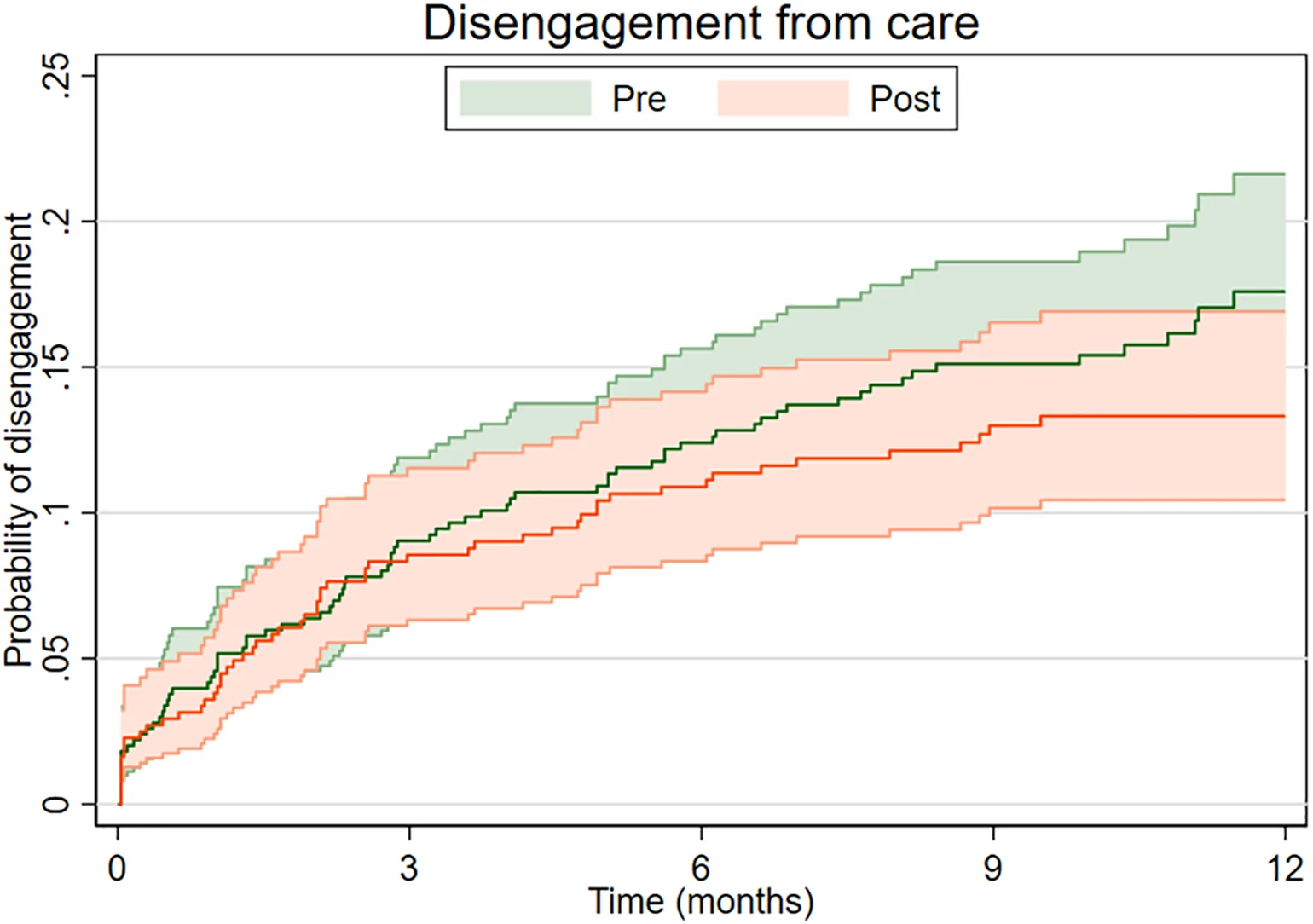
Twelve-month cumulative probability of disengagement from care among clients with advanced HIV disease before and after transition to Ministry of Health-led service delivery.

Kaplan–Meier curves showing the cumulative probability of death over 12 months among clients enrolled in advanced HIV disease (AHD) care during the pre-transition and post-transition periods. Shaded areas represent 95% confidence intervals. Twelve-month mortality declined from 9.4% in the pre-transition period to 5.5% in the post-transition period. In the adjusted Cox proportional hazards model, clients in the post-transition period had a 41% lower hazard of death compared with those in the pre-transition period (adjusted hazard ratio [aHR] = 0.59; 95% CI: 0.37–0.94; **p = 0.026**).

Kaplan–Meier curves showing the cumulative probability of disengagement from care over 12 months among clients enrolled in AHD care during the pre-transition and post-transition periods. Shaded areas represent 95% confidence intervals. There was no statistically significant difference in disengagement from care between the pre-transition and post-transition periods (hazard ratio [HR] = 0.86; 95% CI: 0.62–1.20; **p = 0.384**), based on the Cox proportional hazards model.

## Discussion

In this retrospective cohort study evaluating the phased transition of an AHD care model from EGPAF-led delivery to full MoH stewardship across three districts in Central Malawi, we observed a marked shift towards earlier enrolment and improved outcomes. Patients entering care in the post-transition phase presented with fewer severe opportunistic conditions, including a lower prevalence of severe weight loss and candidiasis, and experienced reduced mortality compared with the NGO-led phase [11]. These findings suggest that decentralised, government-led models can not only sustain but potentially enhance program performance when supported by effective integration into routine health systems [13]. They also align with global evidence underscoring that earlier diagnosis, streamlined service delivery, and program ownership by the MoH are critical determinants of survival for people with AHD [14].

The observed decline in baseline CD4 testing coverage post-transition signals a critical vulnerability in diagnostic infrastructure when donor-supported systems are integrated into routine care. However, the associated shift toward higher median CD4 counts and increased outpatient initiation suggests that case-finding improved via earlier clinical engagement, even as laboratory capacity contracted. This contrast between weakened diagnostics and more proactive care delivery is not unique; studies from low-income settings report that donor transition can erode diagnostic quality, supply chains, and service comprehensiveness, despite preserving service volume. Sustaining gains in HIV care during transition thus demands deliberate support for laboratory systems and innovative strategies, such as affordable point-of-care CD4 technologies, which maintain immunological staging capacity even in decentralized settings. Embedding such diagnostics alongside structured capacity-building and mentorship may facilitate early identification of AHD, preserve the benefits of outpatient care, and ultimately support sustainable AHD responses under government stewardship. The decline in CD4 testing coverage post-transition is attributable to a combination of operational factors. During the pre-transition phase, EGPAF maintained a parallel reagent procurement pipeline and dedicated laboratory mentorship for point-of-care CD4 testing at hub facilities. Following transition to MoH stewardship, CD4 reagents were integrated into the national laboratory supply chain; however, intermittent stockouts and procurement delays within the national system reduced testing availability, particularly at spoke sites. Equipment maintenance challenges and reduced availability of trained laboratory personnel at decentralized facilities further compounded this decline. These challenges were partially anticipated during transition planning, and mitigation strategies – including national-level advocacy for CD4 reagent prioritization and the integration of CD4 into routine viral load testing platforms – were recommended; however, implementation was incomplete in the early post-transition period. The shift toward WHO staging as the predominant AHD eligibility criterion, while reflecting a pragmatic response to reduced laboratory capacity, carries programmatic risks. WHO clinical staging depends on the recognition of specific stage 3 and 4 conditions by frontline providers, and its sensitivity for identifying immunocompromised patients without overt clinical signs is limited compared to CD4-based diagnosis. Consequently, some clients with CD4 counts below 200 cells/mm³ but without manifest WHO stage 3 or 4 conditions may have been missed post-transition, potentially contributing to under-identification of AHD. Strengthening CD4 testing capacity therefore remains an essential priority for accurate case identification, even as clinical staging is reinforced. [15].

The decline in candidiasis and severe weight loss among clients enrolled post-transition compared to pre-transition signals earlier engagement in AHD care and a shift toward less advanced disease at presentation. These findings align with global evidence that decentralised and government-led models, when coupled with rapid ART initiation and systematic screening, can reduce the burden of severe opportunistic conditions [2,16]. However, the persistent presence of cryptococcal meningitis highlights an enduring gap in diagnostic and preventive coverage. Despite WHO recommendations for systematic CrAg screening and pre-emptive antifungal therapy, implementation remains inconsistent in routine settings, with missed opportunities for mortality prevention [2,17]. The concurrent rise in bacterial infections post-transition further reflects weaknesses in antimicrobial stewardship and limited microbiological diagnostic capacity. Together, these trends underscore that while program transition has facilitated earlier and less severe disease presentation, sustaining gains requires deliberate investment in laboratory strengthening and the full implementation of the AHD package, including CrAg screening and integrated infection management strategies, to prevent reversals in progress.

The decrease in mortality with aHR of 0.59 and 0.58 in Cox and Fine–Gray models, respectively, demonstrates the critical value of structured capacity-building and government-led mentorship in stabilizing high-risk AHD cohorts. Notably, this mortality reduction occurred without a corresponding decline in retention, indicating that strengthened clinical outcomes were not offset by increased disengagement from care. These findings are consistent with an expanding body of evidence showing that when complex HIV interventions are transitioned into national health systems, outcomes improve through institutionalized mentoring, workforce strengthening, and integrated service delivery [9,14,18,19]. Moreover, embedding AHD management into government platforms aligns with implementation research that underscores sustainability as a function of local ownership, capacity transfer, and system integration [14]. Together, these results affirm that a government-led AHD strategy, supported by deliberate capacity-building and robust follow-up systems, can achieve measurable survival benefits while laying the foundation for long-term program sustainability.

However, the observed decline in TPT uptake from 46% to 32% during the transition period is concerning, as it highlights how programmatic realignments can inadvertently disrupt continuity of preventive care. Even though reductions in eligibility mirrored declines in diagnostic uptake, this pattern exposes the inherent fragility of TB prevention services in decentralized, transitioning settings. Implementation research underscores that maintaining TPT delivery during stewardship transitions demands robust systems for commodity forecasting, integrated monitoring, and proactive follow-up mechanisms; without these, preventive gains quickly erode Operationally, the decline in TPT uptake post-transition reflects several intersecting system-level factors. First, isoniazid stock availability at facility level became less consistent following transition to national supply chain management, with intermittent stockouts reported at several spoke facilities. Second, the reduction in CD4 testing coverage directly reduced the pool of patients formally identified as TPT-eligible according to national AHD protocols, which require CD4 confirmation for eligibility determination. Third, reduced frequency of EGPAF clinical mentorship visits meant that reminder systems and case-based coaching on TPT initiation – previously provided by partner-embedded mentors – were no longer systematically in place. These operational realities underscore the need for anticipatory transition planning that explicitly addresses TPT supply chain security, eligibility determination under reduced laboratory capacity, and integration of preventive therapy promotion into MoH-led supervision systems. [20–22]. Importantly, safeguards such as task-sharing with community health workers, home-based initiation and follow-up, and integration of TPT into broader primary care platforms have demonstrated effectiveness in preserving preventive coverage in decentralized contexts. As such, national policies must prioritize embedding these systemic supports, particularly during program transitions to sustain progress in AHD interventions and protect the downstream impact on HIV-related mortality [12]. From a health systems perspective, our transition model, with comprehensive matrices covering leadership, procurement, training, monitoring, SOPs, and community linkage, provides a replicable blueprint for program ownership by the governments. Unlike short-term donor-driven projects with limited institutionalization, our phased approach yielded sustained coverage of essential services post-handover, evidenced by mortality benefit supporting calls for integrating evidence generation and co-accountability during handovers.

Several limitations warrant acknowledgment. The observational design precludes causal inference. Documentation gaps and AHD-related supply pipelines, particularly CD4 usage, may have biased baseline AHD definitions. Additionally, we did not quantify costs or measure intervention fidelity, both of which are important given resource constraints in low-income settings. Despite this, our large real-world dataset, rigorous 12-month follow-up, and stratified survival and competing-risk analyses strengthen the credibility and policy relevance of our findings. Additional limitations include: [1] differential data abstraction timing between phases, which may have affected outcome ascertainment completeness, particularly for longer follow-up intervals in the post-transition cohort; [2] the decline in CD4 testing coverage post-transition means that AHD eligibility criteria shifted across phases, limiting direct comparability of enrolled populations and potentially introducing differential misclassification bias; [3] we were unable to conduct disaggregated analyses by sex or age group for survival outcomes due to limited statistical power in subgroups, and therefore equity-related differences in outcomes by these characteristics remain unexplored; [4] differences in baseline characteristics between cohorts – including age distribution, inpatient enrolment, ART exposure, and eligibility criterion – represent potential residual confounders that multivariable adjustment may not have fully addressed; and [5] facility-level heterogeneity in transition pace and fidelity was not formally measured. Future evaluations should incorporate sensitivity analyses adjusting for diagnostic coverage differences and explore equity dimensions through sex- and age-disaggregated outcome reporting.

In conclusion, this study demonstrates that a structured and phased transition of AHD services to national stewardship can deliver sustained mortality reductions without eroding retention, underscoring the feasibility of embedding complex care models within routine health systems. Success was contingent on deliberate capacity-building, robust monitoring, and high-level stakeholder alignment, critical pillars for resilient health system integration. These findings are directly relevant to global HIV strategies seeking to re-centre program ownership within MoH, advancing both sustainability and equity in line with calls to “decolonise” global health. Future priorities must include rigorous evaluation of cost-effectiveness, fidelity to evidence-based clinical algorithms, and scalable delivery models, ensuring that the gains demonstrated here can be replicated across sub-Saharan Africa and other high-burden settings. However, these gains must be understood alongside the significant challenges that emerged: the marked decline in CD4 testing coverage, the reduction in TPT uptake, and the shift away from laboratory-confirmed AHD eligibility represent programmatic vulnerabilities that require deliberate action. Policymakers and implementing partners planning similar transitions should not interpret the mortality benefit observed here as evidence that diagnostic and preventive care gaps are inconsequential; rather, mortality reduction likely occurred despite – not because of – these gaps, due to sustained clinical mentorship and strong health worker capacity built during the pre-transition phase. Transitions that do not safeguard diagnostic infrastructure and preventive therapy supply chains risk undermining long-term program sustainability. These findings offer actionable lessons for WHO, UNAIDS, and national HIV programs seeking to institutionalize AHD care: a phased, domain-specific transition model supported by co-accountable governance structures, national laboratory strengthening, and integrated TPT delivery can protect patient outcomes while advancing program ownership. Replication of this model across sub-Saharan Africa should be accompanied by prospective monitoring systems capable of capturing the differential impact of transition on equity, diagnostic coverage, and preventive care.
