# Supplementary material for: Impact of a phased transition model on Advanced HIV Disease outcomes: A pre- and post-implementation evaluation study in Malawi
Source: PLOS Glob Public Health. 2026 Oct 23;6(10):e0006348. doi: 10.1371/journal.pgph.0006348 (PMC13693884; doi:10.1371/journal.pgph.0006348)
Supplement: Supplementary file 1 [file pgph.0006348.s002.docx]

15 July 2026

The Editors

**PLOS Global Public Health**

Dear Editors,

We are grateful to the Editors and both reviewers for their thorough and constructive critique of our manuscript entitled "Impact of a Phased Transition Model on Advanced HIV Disease Outcomes: A Pre- and Post-Implementation Evaluation Study in Malawi" (Manuscript ID: PGPH-D-25-XXXXX). We have carefully considered all comments and have made substantial revisions to address each point raised. We believe the manuscript has been significantly strengthened as a result.

Below, we provide a point-by-point response to each reviewer comment. For clarity, reviewer comments are presented in italics and our responses follow in standard text. Where applicable, revised manuscript text is shown in a shaded box.

We hope that our revisions adequately address the concerns raised and that the manuscript is now suitable for publication in PLOS Global Public Health.

Yours sincerely,

Thulani Maphosa

On behalf of all co-authors

Elizabeth Glaser Pediatric AIDS Foundation, Lilongwe, Malawi

Email: tmaphosa@pedaids.org

**REVIEWER 1 — Dr. Judith Kose Otieno**

We thank Reviewer 1 for their positive assessment of the manuscript and for raising specific, constructive points that have materially improved the work. We address each comment below.

***Comment 1.1 – Data collection process***

*Please specify who collected the data during both pre- and post-transition phases, and whether they received standardized training or mentorship. Were the same cadres of data collectors involved across phases?*

**Response:**

Thank you for this important methodological point. We have revised the Data Collection and Management section to clarify that data abstraction during both phases was performed by trained research assistants who received standardised training on AHD register completion and data abstraction protocols prior to data collection. We also confirm that the same cadres of data collectors were involved across both phases to ensure consistency.

**Revised manuscript text:**

Data abstraction during both pre- and post-transition phases was performed by trained research assistants who received standardized training on AHD register completion and data abstraction protocols prior to data collection. The same cadres of data collectors were involved across both phases to ensure consistency. [Methods – Data Collection and Management]

***Comment 1.2 – Timing of data abstraction***

*Pre-transition data collection began one month before the end of the period, while post-transition data collection started 10 months before the end. Please explain the rationale for this difference and whether it may have influenced data completeness or quality.*

**Response:**

We have addressed this directly in the revised Methods section. Pre-transition data collection commenced one month before the close of the pre-transition period to enable timely data lock, whereas post-transition data collection began ten months before the end of the post-transition period to allow sufficient follow-up time for 12-month outcome ascertainment. This difference reflects the study's longitudinal design requirements: the post-transition cohort required earlier abstraction to capture complete 12-month follow-up within the evaluation window. We have also added this as an explicit limitation in the revised Limitations paragraph, noting that differential abstraction timing may have affected outcome ascertainment completeness.

***Comment 1.3 – Reasons for reduced CD4 testing coverage and implications for programming***

*Please elaborate on the reasons for reduced CD4 access — was this due to procurement challenges, supply chain disruptions, or reduced prioritization? Was this anticipated during transition planning? What are the consequences of relying more heavily on WHO staging? Could reduced CD4 coverage lead to under-diagnosis of AHD clients?*

**Response:**

We thank the reviewer for raising this critical point. We have substantially expanded the Discussion section to address the operational drivers of the CD4 decline and its programmatic implications. In brief, the decline reflects a combination of factors: integration of CD4 reagents into the national supply chain (with resultant stockouts), equipment maintenance challenges, and reduced availability of trained laboratory personnel at spoke facilities. These challenges were partially anticipated, and mitigation strategies were recommended but incompletely implemented during the early post-transition period. We have also discussed the risk that greater reliance on WHO clinical staging may result in under-identification of immunocompromised patients without overt clinical signs, and reaffirm that strengthening CD4 testing capacity remains essential. These points are addressed both in the Discussion and in the expanded Limitations section.

***Comment 1.4 – WHO stage 3 and 4 conditions post-transition***

*The manuscript should clarify which WHO stage 3 and 4 conditions were most frequently identified post-transition. For example, TB and oral candidiasis diagnoses declined, while bacterial infections increased. This discrepancy warrants further explanation.*

**Response:**

We agree and have added clarification in the Results section. Among WHO stage 3 and 4 defining conditions documented post-transition, pulmonary TB remained the most common, followed by severe bacterial infections and severe pneumonia; oral candidiasis, among the most frequently recorded stage 3 conditions pre-transition, was absent post-transition. We discuss possible explanations for this shift — including changes in provider recognition patterns under a less-intensively mentored system — in the Discussion.

***Comment 1.5 – OI detection and alternative interpretation***

*The discussion states that patients presented with fewer opportunistic conditions post-transition. However, this could also reflect reduced screening sensitivity rather than true declines. Effective programs should detect OIs if present. Please consider this alternative interpretation.*

**Response:**

This is an astute and important observation. We have revised both the Results and Discussion sections to explicitly acknowledge this alternative interpretation. We note that the overall decline in diagnosed OIs should be interpreted cautiously: it may reflect genuine improvement in clinical status at presentation (consistent with earlier enrolment and less advanced disease), but may also partly reflect reduced screening sensitivity in settings where OI-specific protocols were applied less consistently without intensive partner mentorship.

***Comment 1.6 – TPT uptake decline***

*The decline in TB preventive therapy uptake post-transition is concerning. Was this due to procurement or supply chain challenges? Please provide more detail on systemic factors that may have contributed.*

**Response:**

We have added a dedicated paragraph in the Discussion providing detailed operational context for the TPT decline. Specifically, we identify three intersecting factors: (1) intermittent isoniazid stockouts following transition to national supply chain management; (2) reduced CD4 testing coverage directly reducing the pool of formally identified TPT-eligible patients, as national AHD protocols require CD4 confirmation for eligibility determination; and (3) reduced frequency of EGPAF clinical mentorship visits, which previously provided systematic reminder and coaching systems for TPT initiation.

***Comment 1.7 – Equity considerations***

*Were there differences in outcomes by age, sex, or facility type? Disaggregated analysis could strengthen the discussion on equity and inform targeted interventions.*

**Response:**

We acknowledge this important gap. Disaggregated survival analyses by sex and age group were not conducted due to limited statistical power in subgroups, and we have added this as an explicit limitation in the revised Limitations section. We recommend that future evaluations incorporate prospective equity monitoring with sex- and age-disaggregated outcome reporting, and we have included this recommendation in the expanded Conclusion.

***Comment 1.8 – Policy implications for other countries***

*The findings have clear relevance for other high-burden countries considering similar transitions. Please highlight how lessons from Malawi can inform broader WHO or UNAIDS guidance on AHD programming.*

**Response:**

We have substantially expanded the Conclusion to provide explicit policy implications for WHO, UNAIDS, and national HIV programs planning similar transitions. Specifically, we highlight that the phased, domain-specific transition model — supported by co-accountable governance, national laboratory strengthening, and integrated TPT delivery — offers actionable lessons for other high-burden settings. We also caution that the mortality benefit observed should not be interpreted as evidence that diagnostic and preventive care gaps are inconsequential, and emphasise that future transition planning must explicitly safeguard CD4 infrastructure and preventive therapy supply chains.

***Comment 1.9 – Limitations section***

*Strengthen the limitations section by explicitly acknowledging potential biases from data collection timing, reduced diagnostic coverage, and reliance on retrospective records. Consider adding a sensitivity analysis.*

**Response:**

We have substantially expanded the Limitations section to explicitly acknowledge: (1) differential data abstraction timing between phases; (2) diagnostic coverage bias arising from the CD4 decline; (3) the absence of equity-disaggregated analyses; (4) residual confounding from baseline differences in cohort characteristics; and (5) facility-level heterogeneity in transition pace and fidelity. Regarding sensitivity analyses, we acknowledge this as a recommended priority for future work and have noted it in the Limitations as an area requiring additional evaluation, recognising it is beyond the scope of this retrospective cohort study with the current dataset.

**REVIEWER 2 — Anonymous**

We thank Reviewer 2 for their detailed and rigorous critique. We agree with their overall assessment that the manuscript has strong scientific merit and have made major revisions to address all concerns raised.

***Comment 2.1 – Pre-post study design, baseline differences, and causal interpretation***

*The principal concern relates to interpretation of the findings in light of the observational pre-post study design. The pre- and post-transition cohorts differ substantially at baseline. The manuscript occasionally overstates causality. Conclusions should be framed more cautiously.*

**Response:**

We fully agree with this concern and have revised the manuscript throughout to moderate the language and frame findings as associations rather than causal effects. In particular, the Discussion and Conclusion now explicitly note that the observational design precludes causal inference, and we have contextualised the mortality reduction in light of the substantial baseline differences between cohorts. The revised Conclusion offers a balanced perspective that acknowledges both the programme's successes and its vulnerabilities, without overstating causal attribution to the transition.

***Comment 2.2 – Statistical methods: model specification, missing data, proportional hazards, clustering, sensitivity analyses***

*The statistical methods section lacks sufficient methodological detail for reproducibility. The authors should clearly specify: variables included in multivariable models; model-building strategy; handling of missing data; assessment of proportional hazards assumptions; whether clustering at facility level was accounted for; and whether sensitivity analyses were conducted.*

**Response:**

Thank you for this important critique. We have substantially expanded the Statistical Analysis section to address each of these points. Specifically: (1) Multivariable models were built using a purposive variable selection approach including clinically and programmatically relevant covariates specified a priori: transition phase, age group, sex, ART treatment status, inpatient enrolment status, and AHD eligibility criterion (CD4 vs. WHO staging). (2) The proportional hazards assumption was evaluated using Schoenfeld residuals and complementary log-log plots, with no significant violations identified. (3) Missing data were handled using complete-case analysis, given that missingness was predominantly in laboratory variables (notably CD4 count) that were themselves outcome indicators. (4) Facility-level clustering was accounted for by stratifying Cox models by facility. Regarding sensitivity analyses, we acknowledge this as a limitation and have recommended it as a priority for future work.

**Revised manuscript text:**

Multivariable models were built using a purposive selection approach, including variables identified a priori as clinically or programmatically relevant: transition phase, age group, sex, ART treatment status, inpatient enrollment status, AHD eligibility criterion (CD4 vs. WHO staging), and WHO stage. The proportional hazards assumption was evaluated graphically using Schoenfeld residuals and complementary log-log plots; no significant violations were identified. Missing data were handled using complete-case analysis given that missingness was predominantly in laboratory variables (notably CD4 count), which were themselves outcome indicators. Facility-level clustering was accounted for by including facility as a stratification variable in Cox models, given the cluster-based design of the study. [Methods – Statistical Analysis]

***Comment 2.3 – CD4 testing coverage decline: operational explanation***

*The substantial decline in CD4 testing coverage deserves deeper operational explanation. The authors should clarify whether this reflects national policy changes, diagnostic stockouts, equipment limitations, reduced donor support, laboratory system challenges, or other structural factors.*

**Response:**

We fully agree and have substantially expanded the Discussion to address this. As described in the revised manuscript, the decline reflects several intersecting operational factors: (1) reagent stockouts and procurement delays following integration into the national supply chain; (2) equipment maintenance challenges; and (3) reduced availability of trained laboratory personnel at decentralised spoke facilities. We note that these challenges were partially anticipated and that mitigation strategies were recommended but incompletely implemented during the early post-transition period. We have also addressed the implications of shifting toward WHO staging as the predominant eligibility criterion, including the risk of under-identification of AHD clients without overt clinical signs.

***Comment 2.4 – Exclusion of 2022 transition year***

*The exclusion of the 2022 transition year from the analysis requires additional justification and explanation. Readers would benefit from clearer rationale and a brief description of transition dynamics during that period.*

**Response:**

We thank the reviewer for raising this point. We have added a detailed justification in the Study Population and Eligibility Criteria section. In brief, the 2022 year was excluded a priori to avoid mixing exposure states: during this period, program leadership was jointly shared between EGPAF and the MoH, and the pace of responsibility transfer varied substantially across the 22 facilities and nine transition domains. Including clients enrolled during this period would introduce heterogeneous and unmeasured exposure, complicating interpretation of phase-specific outcomes. We also briefly describe the key transition activities that occurred during 2022, including structured co-mentorship, joint SOP revision, and progressive handover of commodity procurement.

**Revised manuscript text:**

The decision to exclude the 2022 transition year was made a priori to avoid mixing exposure states within a single analytical cohort. During 2022, program leadership was jointly shared between EGPAF and the MoH, and the pace and completeness of responsibility transfer varied substantially across the 22 facilities and nine transition domains (Table 1). Including clients enrolled during this period would introduce heterogeneous and unmeasured exposure, complicating the interpretation of phase-specific outcomes... [Methods – Study Population and Eligibility Criteria]

***Comment 2.5 – English language editing***

*The manuscript would benefit from substantial English language editing. There are several grammatical errors, awkward sentence constructions, inconsistent terminology, formatting inconsistencies, and typographical issues throughout.*

**Response:**

We thank the reviewer for this observation. We have carefully reviewed and edited the entire manuscript for grammatical accuracy, sentence clarity, and terminological consistency. Key corrections include: standardisation of spelling (e.g., 'enrolment', 'organised'), correction of subject-verb agreement errors, simplification of awkwardly constructed sentences in the Discussion, and removal of typographical inconsistencies in tables and section headings. We are confident the revised manuscript reads clearly and meets PLOS Global Public Health standards for written English.

***Comment 2.6 – Data availability statement***

*The current statement does not fully satisfy PLOS data-sharing expectations. The authors should provide a clearer explanation regarding access procedures and whether a de-identified dataset can be made available through a controlled-access mechanism.*

**Response:**

We have substantially revised the Data Availability statement to address PLOS data-sharing requirements. The revised statement explains that full public release of individual-level patient data is not permitted under the conditions of ethical approval from the National Health Sciences Research Committee of Malawi (NHSRC) and the Advarra Institutional Review Board (protocol number Pro00057717), as the data contain potentially identifiable health information. We confirm, however, that researchers may request access to a de-identified dataset by submitting a formal data access request to the corresponding author, subject to approval by the NHSRC and the EGPAF data governance committee. All aggregate-level summary data presented in this manuscript are fully available within the article and its tables.

**Revised manuscript text:**

Data are available on reasonable request. Data may be obtained from a third party and is not publicly available. The data supporting this study's findings were abstracted from routine clinical records held by the Malawi Ministry of Health and Elizabeth Glaser Pediatric AIDS Foundation (EGPAF). Full public release of individual-level patient data is not permitted under the conditions of ethical approval granted by the National Health Sciences Research Committee of Malawi (NHSRC) and the Advarra Institutional Review Board (protocol number Pro00057717), as these data contain potentially identifiable health information. Researchers requesting access to a de-identified dataset for purposes of replication or secondary analysis may submit a formal data access request to the corresponding author (tmaphosa@pedaids.org), subject to approval by the NHSRC and EGPAF data governance committee. Aggregate-level summary data presented in this manuscript are fully available within the article and its tables. [Declarations – Availability of data and materials]

**Summary of Revisions**

The table below summarises all revisions made in response to reviewer comments and their locations in the revised manuscript.

| **#** | **Reviewer Comment** | **Action Taken** | **Manuscript Location** |
| --- | --- | --- | --- |
| **R1.1** | Who collected data; training; cadres | Clarified that trained research assistants with standardised training conducted abstraction across both phases using the same cadres | Methods – Data Collection |
| **R1.2** | Timing of data abstraction | Explained rationale for differential abstraction timing; added as explicit limitation | Methods – Data Collection; Limitations |
| **R1.3** | Reasons for CD4 decline and AHD under-diagnosis risk | Added operational explanation (stockouts, equipment, personnel); discussed implications of shift to WHO staging | Discussion; Limitations |
| **R1.4** | Which WHO stage 3/4 conditions post-transition | Added clarification of most frequent conditions; discussed candidiasis absence and rise in bacterial infections | Results; Discussion |
| **R1.5** | OI screening sensitivity as alternative explanation | Added cautionary interpretation that OI decline may partly reflect reduced screening sensitivity | Results; Discussion |
| **R1.6** | Systemic factors for TPT decline | Added paragraph on isoniazid stockouts, reduced CD4 eligibility determination, and reduced mentorship | Discussion |
| **R1.7** | Equity by age/sex/facility type | Acknowledged as limitation; recommended future disaggregated analyses | Limitations; Conclusion |
| **R1.8** | Policy implications for WHO/UNAIDS | Expanded Conclusion with explicit policy messages for other high-burden countries | Conclusion |
| **R1.9** | Strengthen limitations section; sensitivity analysis | Substantially expanded to address 5 specific bias domains; sensitivity analysis recommended as future work | Limitations |
| **R2.1** | Observational design; baseline differences; causal language | Moderated language throughout; conclusions reframed as associations; balanced Conclusion added | Throughout; Conclusion |
| **R2.2** | Statistical model specification; missing data; PH assumption; clustering | Added full specification of multivariable models, model-building strategy, missing data handling, PH testing, and facility stratification | Methods – Statistical Analysis |
| **R2.3** | CD4 decline: operational explanation | Expanded Discussion (same as R1.3 above) | Discussion |
| **R2.4** | Justification for excluding 2022 | Added detailed justification and description of 2022 transition dynamics | Methods – Study Population |
| **R2.5** | English language editing | Comprehensive grammar, style, and consistency editing throughout | Throughout |
| **R2.6** | Data availability statement | Revised to explain controlled-access mechanism, ethical basis for restrictions, and contact route for data requests | Declarations |

We thank the editorial team and reviewers once more for their time and expertise. We look forward to the editors' decision.

Sincerely,

Thulani Maphosa, and co-authors
