# Supplementary material for: Impact of a phased transition model on Advanced HIV Disease outcomes: A pre- and post-implementation evaluation study in Malawi
Source: PLOS Glob Public Health. 2026 Oct 23;6(10):e0006348. doi: 10.1371/journal.pgph.0006348 (PMC13693884; doi:10.1371/journal.pgph.0006348)
Supplement: Peer review history

# Peer review: Decision Letter 0

**DOI:** 10.1371/journal.pgph.0006348.r001

**Authors:** Everton Falcão de Oliveira

24 May 2026

PGPH-D-26-01296

Impact of a Phased Transition Model on Advanced HIV Disease Outcomes: A Pre- and Post-Implementation Evaluation Study in Malawi

PLOS Global Public Health

Dear Dr. Maphosa,

Thank you for submitting your manuscript to PLOS Global Public Health. After careful consideration, we feel that it has merit but does not fully meet PLOS Global Public Health’s publication criteria as it currently stands. Therefore, we invite you to submit a revised version of the manuscript that addresses the points raised during the review process.

Please submit your revised manuscript by Jul 23 2026 11:59PM. If you will need more time than this to complete your revisions, please reply to this message or contact the journal office at globalpubhealth@plos.org. When you're ready to submit your revision, log on to https://www.editorialmanager.com/pgph/ and select the 'Submissions Needing Revision' folder to locate your manuscript file.

Please include the following items when submitting your revised manuscript:

- A letter that responds to each point raised by the editor and reviewer(s). You should upload this letter as a separate file labeled 'Response to Reviewers'.
- A marked-up copy of your manuscript that highlights changes made to the original version. You should upload this as a separate file labeled 'Revised Manuscript with Track Changes'.
- An unmarked version of your revised paper without tracked changes. You should upload this as a separate file labeled 'Manuscript'.

Guidelines for resubmitting your figure files are available below the reviewer comments at the end of this letter.

As the corresponding author, your ORCID iD is verified in the submission system and will appear in the published article. PLOS supports the use of ORCID, and we encourage all coauthors to register for an ORCID iD and use it as well. Please encourage your coauthors to verify their ORCID iD within the submission system before final acceptance, as unverified ORCID iDs will not appear in the published article. *Only*  the individual author can complete the verification step; PLOS staff *cannot*  verify ORCID iDs on behalf of authors.

We look forward to receiving your revised manuscript.

Kind regards,

Everton Falcão de Oliveira, Ph.D

Academic Editor

PLOS Global Public Health

Journal Requirements:

1. Please send a completed 'Competing Interests' statement, including any COIs declared by your co-authors. If you have no competing interests to declare, please state "The authors have declared that no competing interests exist". Otherwise please declare all competing interests beginning with the statement "I have read the journal’s policy and the authors of this manuscript have the following competing interests:"

2. Please ensure that your Ethics Statement is available in its entirety at the beginning of your Methods section, under a subheading 'Ethics Statement'.

3. We ask that a manuscript source file is provided at Revision. Please upload your manuscript file as a .doc, .docx, .rtf or .tex.

4. Please provide separate figure files in .tif or .eps format.

For more information about figure files please see our guidelines:  LINK

https://journals.plos.org/globalpublichealth/s/figures

https://journals.plos.org/globalpublichealth/s/figures#loc-file-requirements

5. Your manuscript is missing the following sections: Introduction. Please ensure these are present, and in the correct order, and that any references to subheadings in your main text are correct. An outline of the required sections can be consulted in our submission guidelines here: [LINK]

https://journals.plos.org/globalpublichealth/s/submission-guidelines#loc-parts-of-a-submission

If the reviewer comments include a recommendation to cite specific previously published works, please review and evaluate these publications to determine whether they are relevant and should be cited. There is no requirement to cite these works unless the editor has indicated otherwise.

Additional Editor Comments (if provided):

[Note: HTML markup is below. Please do not edit.]

Reviewers' comments:

Reviewer’s Responses to Questions

**Comments to the Author**

1. Does this manuscript meet PLOS Global Public Health’s publication criteria?

Reviewer #1: Yes

Reviewer #2: Partly

**********

2. Has the statistical analysis been performed appropriately and rigorously?

Reviewer #1: Yes

Reviewer #2: Yes

**********

3. Have the authors made all data underlying the findings in their manuscript fully available (please refer to the Data Availability Statement at the start of the manuscript PDF file)?

The PLOS Data policy

Reviewer #1: Yes

Reviewer #2: No

**********

4. Is the manuscript presented in an intelligible fashion and written in standard English?

Reviewer #1: Yes

Reviewer #2: No

**********

Reviewer #1: Thank you for the opportunity to review this manuscript. It addresses a highly relevant and timely topic, the impact of transitioning advanced HIV disease (AHD) services to Ministry of Health leadership in Malawi, particularly in the context of declining official development assistance (ODA) in the Global south. The study contributes important evidence on sustainability and national ownership of HIV programs.

Please see my detailed comments below:

Study design and data collection

• Clarify data collection process: Please specify who collected the data during both pre- and post-transition phases, and whether they received standardized training or mentorship. Were the same cadres of data collectors involved across phases? This is critical for assessing consistency and potential bias.

• Timing of data abstraction: Pre-transition data collection began one month before the end of the period, while post-transition data collection started 10 months before the end. Please explain the rationale for this difference and discuss whether it may have influenced data completeness or quality.

Eligibility criteria and diagnostics

• Shift in AHD eligibility: The manuscript notes that WHO staging was more frequently used post-transition, while CD4 testing coverage declined. Please elaborate on the reasons for reduced CD4 access, was this due to procurement challenges, supply chain disruptions, or reduced prioritization? Was this anticipated during transition planning?

• Implications for programming: What are the consequences of relying more heavily on WHO staging? Could reduced CD4 coverage lead to under-diagnosis of AHD clients? Please discuss the potential programmatic losses and whether strengthening CD4 testing remains essential for accurate case identification.

• WHO stage conditions: The manuscript should clarify which WHO stage 3 and 4 conditions were most frequently identified post-transition. For example, TB and oral candidiasis diagnoses declined, while bacterial infections increased. This discrepancy warrants further explanation.

Opportunistic infections and preventive therapy

• OI detection: The discussion states that patients presented with fewer opportunistic conditions post-transition. However, this could also reflect reduced screening sensitivity rather than true declines. Effective programs should detect OIs if present. Please consider this alternative interpretation.

• TPT uptake: The decline in TB preventive therapy uptake post-transition is concerning. Was this due to procurement or supply chain challenges? Please provide more detail on systemic factors that may have contributed.

Interpretation and discussion

• Program sustainability: While mortality declined, the reduction in CD4 testing and TPT uptake highlights vulnerabilities in sustaining comprehensive AHD care. Please expand on how these gaps could be addressed in future nationally-led models.

• Equity considerations: Were there differences in outcomes by age, sex, or facility type? Disaggregated analysis could strengthen the discussion on equity and inform targeted interventions.

• Policy implications: The findings have clear relevance for other high-burden countries considering similar transitions. Please highlight how lessons from Malawi can inform broader WHO or UNAIDS guidance on AHD programming.

Additional suggestions

• Strengthen the limitations section by explicitly acknowledging potential biases from data collection timing, reduced diagnostic coverage, and reliance on retrospective records.

• Consider adding a sensitivity analysis to test whether mortality declines remain robust when adjusting for diagnostic coverage differences.

• Expand the conclusion to emphasize both the successes (mortality reduction, sustained retention) and the risks (diagnostic gaps, preventive therapy decline), providing a balanced perspective for policymakers.

Reviewer #2: This manuscript addresses an important and highly relevant topic in global HIV implementation science: the transition of Advanced HIV Disease (AHD) services from donor-supported implementation to Ministry of Health (MoH) ownership in Malawi. The study contributes valuable real-world evidence regarding sustainability, health systems strengthening, and nationally led HIV service delivery in high-burden settings. The use of routine programmatic data from 22 facilities, combined with survival and competing-risk analyses, represents a meaningful contribution to the literature on AHD outcomes and transition science.

The manuscript is generally well structured and demonstrates substantial public health relevance. The findings suggesting reduced mortality following transition to MoH stewardship are important and potentially impactful for HIV policy and implementation programs across sub-Saharan Africa. The transition framework presented across multiple operational domains is also a notable strength and may serve as a useful model for similar programs.

However, the manuscript requires major revision before it can be considered for publication.

The principal concern relates to interpretation of the findings in light of the observational pre-post study design. The pre- and post-transition cohorts differ substantially at baseline with respect to age distribution, inpatient enrollment, ART exposure, WHO staging, and CD4 eligibility criteria. These differences introduce potential confounding and limit causal interpretation of the mortality reduction observed. While the analyses demonstrate an association between the transition period and improved outcomes, the manuscript occasionally overstates causality. Conclusions should therefore be moderated and framed more cautiously.

The statistical analysis methods are broadly appropriate, including the use of Kaplan–Meier survival curves, Cox proportional hazards models, and Fine and Gray competing-risk regression. However, the statistical methods section lacks sufficient methodological detail for reproducibility and rigorous interpretation. The authors should clearly specify:

variables included in multivariable models,

model-building strategy,

handling of missing data,

assessment of proportional hazards assumptions,

whether clustering at facility level was accounted for,

and whether sensitivity analyses were conducted.

The substantial decline in CD4 testing coverage post-transition is one of the manuscript’s most important findings and deserves deeper operational explanation. The discussion currently provides only limited speculation regarding this decline. The authors should clarify whether this reflects national policy changes, diagnostic stockouts, equipment limitations, reduced donor support, laboratory system challenges, or other structural factors. Similarly, the decline in TPT uptake requires stronger contextual and operational interpretation linked to implementation realities in Malawi.

The exclusion of the 2022 transition year from the analysis also requires additional justification and explanation. Since the transition phase likely represents the most operationally informative period, readers would benefit from clearer rationale regarding its exclusion and a brief description of transition dynamics during that period.

The manuscript would also benefit from substantial English language editing. Although generally intelligible, there are several grammatical errors, awkward sentence constructions, inconsistent terminology, formatting inconsistencies, and typographical issues throughout the text that should be corrected prior to publication.

Regarding data availability, the current statement does not fully satisfy PLOS data-sharing expectations. While the ethical and legal restrictions are understandable, the authors should provide a clearer explanation regarding access procedures and whether a de-identified dataset can be made available through a controlled-access mechanism.

Despite these concerns, the manuscript has strong scientific merit and considerable policy relevance. With substantial revision to strengthen methodological transparency, improve interpretation, clarify operational findings, and refine language, the study has good potential for publication.

**********

what does this mean?). If published, this will include your full peer review and any attached files.

**Do you want your identity to be public for this peer review?**  If you choose “no”, your identity will remain anonymous but your review may still be made public.

For information about this choice, including consent withdrawal, please see our Privacy Policy

Reviewer #1: **Yes:**  Dr. Judith Kose Otieno

Reviewer #2: No

**********

[NOTE: If reviewer comments were submitted as an attachment file, they will be attached to this email and accessible via the submission site. Please log into your account, locate the manuscript record, and check for the action link "View Attachments". If this link does not appear, there are no attachment files.]

# Author comment: Author response to Decision Letter 1

**DOI:** 10.1371/journal.pgph.0006348.r002

22 Aug 2026

Attachment Submitted filename: Response_to_Reviewers.docx

# Peer review: Decision Letter 1

**DOI:** 10.1371/journal.pgph.0006348.r003

**Authors:** Everton Falcão de Oliveira

21 Sep 2026

Impact of a Phased Transition Model on Advanced HIV Disease Outcomes: A Pre- and Post-Implementation Evaluation Study in Malawi

PGPH-D-26-01296R1

Dear Dr Maphosa,

We are pleased to inform you that your manuscript 'Impact of a Phased Transition Model on Advanced HIV Disease Outcomes: A Pre- and Post-Implementation Evaluation Study in Malawi' has been provisionally accepted for publication in PLOS Global Public Health.

Before your manuscript can be formally accepted you will need to complete some formatting changes, which you will receive in a follow up email. A member of our team will be in touch with a set of requests.

Please note that your manuscript will not be scheduled for publication until you have made the required changes, so a swift response is appreciated.

IMPORTANT: The editorial review process is now complete. PLOS will only permit corrections to spelling, formatting or significant scientific errors from this point onwards. Requests for major changes, or any which affect the scientific understanding of your work, will cause delays to the publication date of your manuscript.

If your institution or institutions have a press office, please notify them about your upcoming paper to help maximize its impact. If they'll be preparing press materials, please inform our press team as soon as possible -- no later than 48 hours after receiving the formal acceptance. Your manuscript will remain under strict press embargo until 2 pm Eastern Time on the date of publication. For more information, please contact globalpubhealth@plos.org.

Thank you again for supporting Open Access publishing; we are looking forward to publishing your work in PLOS Global Public Health.

Best regards,

Everton Falcão de Oliveira, Ph.D

Academic Editor

PLOS Global Public Health

***********************************************************

Reviewer Comments (if any, and for reference):

Reviewer’s Responses to Questions

**Comments to the Author**

Reviewer #1: All comments have been addressed

**********

publication criteria?

Reviewer #1: Yes

**********

3. Has the statistical analysis been performed appropriately and rigorously?

Reviewer #1: Yes

**********

4. Have the authors made all data underlying the findings in their manuscript fully available (please refer to the Data Availability Statement at the start of the manuscript PDF file)?

The PLOS Data policy

Reviewer #1: Yes

**********

5. Is the manuscript presented in an intelligible fashion and written in standard English?

Reviewer #1: Yes

**********

Reviewer #1: Thank you for the revisions made

**********

what does this mean?). If published, this will include your full peer review and any attached files.

**Do you want your identity to be public for this peer review?**  If you choose “no”, your identity will remain anonymous but your review may still be made public.

For information about this choice, including consent withdrawal, please see our Privacy Policy

Reviewer #1: **Yes:**  Dr Judith Kose

**********
